# When Gold is Tarnished: The Impact of Smoker's Breast Milk on Offspring Growth

**DOI:** 10.1016/j.jpedcp.2024.200132

**Published:** 2024-10-26

**Authors:** Elizabeth Yen

**Affiliations:** 1 Tufts University School of Medicine, Boston, MA; 2 Mother Infant Research Institute, Tufts Medical Center, Boston, MA; 3 Tufts Medicine Pediatrics-Boston Children's, Boston, MA

**Keywords:** breast milk, breastfeeding, cigarette smoking, growth trajectory, sex differences

Breast milk is liquid gold. It connects a mother with her baby in an intimate way. Its unique composition (water, fat, proteins, electrolytes, vitamins, minerals, hormones, growth factors, and immune cells) is precisely modulated by maternal and infant characteristics and orchestrated in harmony unparalleled by other forms of nutrition.^1^, ^2^, ^3^ No two breast milk samples are the same.

Beyond the nutritional value in the immediate period after birth, breast milk confers lifelong benefits. The American Academy of Pediatrics (AAP) posted an extensive list in its Policy Statement.^4^ In infants, breast milk reduces the risk of sudden infant death syndrome, overall postnatal and infant mortality, infections, diarrhea, eczema, asthma, obesity, diabetes, and even leukemia. For mothers, breastfeeding reduces their risk of diabetes, hypertension, and various types of cancer (breast, ovarian, endometrium, and thyroid).^4^ In addition to these individual benefits, breast milk also provides transgenerational advantages, likely from the epigenetic changes related to human milk.^5^^,^^6^ As a result, many health agencies, including the AAP, fully endorse breastfeeding.

The AAP recommends *exclusive* breastfeeding in the first six months and *ongoing* breastfeeding until 2 years of age.^4^ Healthy People 2030 targets 42.4% of infants in the US to be exclusively breastfed in the first six months and 54.1% to continue breastfeeding until one year.^7^ Except for a few absolute contraindications to breastfeeding (galactosemia, untreated or unsuppressed HIV, human T-cell lymphotropic virus type I or II infection, untreated brucellosis, suspected or confirmed *Ebolavirus*, and illicit substance use),^4^^,^^8^ breast milk is the ideal and standard nutrition for infants.

Yet, in several understudied conditions, the guideline is not as straightforward. Maternal cigarette smoking is one of them. Exposure to secondhand smoke is linked to increased sudden infant death syndrome, asthma, and respiratory issues.^4^ But recognizing the many benefits of breastfeeding, the AAP states that “breastfeeding mothers should be encouraged strongly to stop smoking and to minimize secondhand smoke” and “if, after counseling, a breastfeeding mother chooses to smoke or vape, she should be advised to minimize her smoking, never smoke while breastfeeding, and never smoke inside the home or car.”^4^ Even with this recommendation, a survey showed that the majority of the mothers still believed that breastfeeding while smoking was not acceptable.^9^ There needs further propagation of what the AAP has recommended all along: yes to breastfeeding, no to smoking, and when smoking cessation is not possible, be judicious about when and where to breastfeed.^4^^,^^10^

In this volume of *The Journal of Pediatrics: Clinical Practice*, Shenassa et al conducted the largest and most detailed study of feeding methods in a setting of postpartum smoking.^11^ As smokers' breast milk has been shown to induce obesity (‘obesogenic’), the authors aimed to study the synergy between feeding method, nicotine exposure, and infant growth at one year. The secondary aim was to examine sex differences. More than 54,000 infants in the Norwegian Mother, Father, and Child Cohort and Medical Birth Registry of Norway born between 1999 and 2008 were included in this study. Mothers who consented before their pregnancy filled out serial questionnaires related to their feeding practice (breast-, formula-, or mixed feeding), smoking status during and after pregnancy (none, light [1-10 cigarettes/day], or heavy [>10 cigarette/day]), and their infants' anthropometric data (weight, length, and weight-for-length *z* scores [WFLZs]).

At birth, infants of heavy smokers were 99 grams lighter and 5 mm shorter than those of nonsmoking mothers. At 1 year, however, infants of heavy smokers had an *additional* weight gain of 172 (95% CI: 132, 211) grams, length of 1.7 (95% CI: 0.08, 2.6) mm, and WFLZ of 0.12 (95% CI: 0.08, 0.15) than those of nonsmokers. These gains were all statistically significant. Joint effect analysis of feeding methods and maternal smoking at one year showed that among babies exclusively breastfed, those breastfed by smokers experienced significantly more rapid growth than those breastfed by nonsmokers. A dose-response relationship was also observed—the heavier mothers smoke while breastfeeding, the more rapidly their infants grow.

To study the interaction between feeding methods and maternal smoking and to determine whether these gains were attributed to smokers' breast milk, the authors used the ‘difference in difference’ method. In this method, any growth difference between smoking-exposed infants and nonexposed infants was attributable to the source of exposure, that is, *not* common between the 2 groups, (ie, breast milk). The authors reported evidence of interaction in infants who were *ever* breastfed by heavy smokers, with a significantly greater length gain in those ever breastfed by smokers (2.8 mm, 95% CI 0.1, 5.6). While these infants also gained weight and WFLZ, these gains were not statistically significant. When stratified by sex, the evidence of interaction was seen in females but not males. Altogether, these results suggest that infants breastfed by mothers with a heavy [>10 cigarette/day] cigarette habit demonstrated a concerning growth trajectory. Hence, the amount of maternal smoking may limit the positive effects of breast milk on infant growth. However, in infants breastfed by light smokers, these gains did not “meaningfully vary from population norms,” as evidenced by the low WFLZ values. The authors concluded that the benefits of breastfeeding far outweigh short-term obesogenic consequences.

Shenassa et al did a comprehensive evaluation of this challenging topic and provided evidence of the adverse effects related to heavy smoking, as well as reassurance to support concurrent breastfeeding and light cigarette smoking. As pointed out by the authors, however, this retrospective study has several drawbacks—selection bias, sample bias, lack of precise measurements and variables, and short duration of follow-up—that limit its interpretation. Additionally, Norway has healthcare profiles that are different from those of other countries, limiting the generalizability of this study to other populations. A study comparing childhood obesity in Norway, Canada, and the US showed that the extent was lower for Norway and Canada and greatest for the US.^12^ Therefore, an insignificant weight gain at one year of age in Norway may set off a completely different and rather substantial weight trajectory in America. As females are at twice the risk of obesity and overweight-related mortality,^13^ the greater interaction seen in females than in males also poses further questions regarding the long-term implications of this sex-specific effect on the greater risks and adverse health outcomes in females. Paralleling the epigenetic changes seen with breast milk, cigarette smoking also alters DNA methylation with its transmission to several generations.^14^^,^^15^ Therefore, while this study did not show adverse growth effects in infants breastfed by light smokers at one year, much more remains unknown and requires further investigation. As Carl Sagan, an American astronomer and planetary scientist, once said, “Absence of evidence is not evidence of absence.”^16^

Breast milk is liquid gold. Pure gold does not tarnish, but the impurity mixed within it does, and it does so with time. Yet even with its tarnish and impurity, it is gold, after all. As more studies are underway to elucidate and answer the unknowns, as a community, we should continue to enforce the value of breast milk yet seize every opportunity to always counsel mothers and families to make the best decision for themselves, their babies, and their future grandchildren. Breastfeeding is good, but smoking is not. One should never lose sight of that.

## Declaration of Competing Interest

The author declares no conflict of interest.
